# Milking-Order Stability and Stabilization After Herd Entry Under Heat Stress: An Observational Study in a Large Dairy Herd

**DOI:** 10.3390/ani16182891

**Published:** 2026-09-14

**Authors:** Yifeng Song, Zhalaga, Muqier, Xiaoping An, Na Liu, Jingwei Qi

**Affiliations:** 1 College of Animal Science, Inner Mongolia Agricultural University, Hohhot 010018, China; 2 Key Laboratory of Smart Animal Husbandry at Universities of Inner Mongolia Autonomous Region/National Center of Technology Innovation for Dairy-Breeding and Production Research Center, Hohhot 010018, China; 3 Inner Mongolia Shengmu High-Tech Farming Company, Limited, Hohhot 010111, China

**Keywords:** heat stress, dairy cows, milking order, herd entry

## Abstract

Heat stress is known to affect dairy cow behavior, but its effects on milking-order patterns and stabilization after herd entry in large commercial herds are not well described. We analyzed 70,491 parlor records from 484 Holstein cows milked three times daily in a free-stall herd to examine how thermal conditions shape milking-order dynamics. Daily intraday milking-order displacement was quantified based on changes in queue position between successive milkings. Cow-specific regressions of milking order on ambient temperature were used to identify clusters of thermal response, and time to reach a stable queue position was evaluated for newly introduced cows according to thermal conditions at herd entry. Results show a modest increase in intraday physical displacement from thermally comfortable to severely hot days, whereas relative displacement did not differ significantly across the four thermal categories. Cows showed consistent individual differences in how their milking position shifted with temperature that were only weakly associated with parity, days in milk, milk yield, or panting score. In an exploratory severe versus non-severe comparison, cows entering the herd under severe thermal conditions had a lower adjusted hazard of reaching the predefined stabilization criterion (HR = 0.62, 95% CI: 0.43–0.90). Collectively, these findings indicate that milking-order patterns extracted from routine parlor records are associated with behavioral responses to thermal conditions. Their management implications, including decisions related to herd entry and cooling, require further experimental evaluation.

## Simple Summary

Heat stress is a major challenge for dairy farms, yet most investigations focus on barn temperature rather than how cows actually behave. In this study, we used routine parlor records from a large commercial herd to describe how heat load changes milking-order patterns and how quickly newly introduced cows settle into a stable place in the queue. Our findings suggest that milking-order measures may provide complementary information about cows’ behavioral responses to thermal conditions and may support future research on herd-entry and cooling management.

## 1. Introduction

Heat stress is now recognized as a major biological and management constraint on intensive dairy systems, as rising ambient temperatures and humidity increasingly exceed the thermoregulatory capacity of high-yielding cows [1]. Beyond the well-characterized reductions in milk yield, reproductive performance, and survival [2,3], heat stress reshapes time budgets, feeding behavior, and social interactions [4,5,6]. These shifts compromise both productivity and welfare at the herd level and challenge conventional approaches to heat-stress detection, which often focus on short-term production losses rather than early behavioral disruption.

In large and intensively managed herds, milking parlor routines impose a strong temporal structure on cows’ daily access to feed, rest, and cooling [7,8]. Across both grazing and housed dairy systems, and in herds milked in either conventional parlors or automatic milking systems, cows are consistently observed to enter for milking in a relatively repeatable sequence, indicating that milking order is a structured, herd-level behavioral pattern rather than random variation [9,10,11]. Consequently, even subtle, systematic deviations in how animals queue and enter the parlor may provide an early, management-relevant signal that individuals are changing their behavioral strategies to cope with environmental challenges, including heat stress [12].

Previous work on milking order has mainly examined the absolute position that cows occupy in the queue and its association with cow-related characteristics. Since the early study by Rathore [13], most studies have identified that higher-yielding cows enter the milking parlor earlier, but this effect can be confounded or even modified by cow-related factors such as parity and lactation stage [8,11]. After adjusting for cow-related factors, Berry and McCarthy [14] still found a weak yet positive partial correlation between milking order and milk yield. More recently, Vargas-Bello-Pérez et al. [15] shifted attention from absolute position to temporal stability, finding that cows with a more stable milking order over time tended to have higher milk yields. This pattern suggests that the stability of milking order may more directly capture a cow’s ability to tolerate and recover from external challenges compared with absolute position. Collectively, previous studies support milking order and especially its stability as a meaningful behavioral phenotype, but they rarely considered heat stress as an influencing factor, leaving unresolved the central question of whether and to what extent heat stress is associated with milking-order dynamics.

Against this backdrop, this study aimed to integrate high-resolution parlor entrance records from a large-scale free-stall herd with environmental indicators to examine milking-order dynamics under different thermal conditions. Specifically, this study had three objectives. First, we examined whether greater daily heat load was associated with greater intraday milking-order displacement. Second, we assessed whether cows differed in the association between ambient temperature and milking-order position. Third, we evaluated whether the thermal condition at herd entry was associated with the number of milking events required to reach a predefined stabilization criterion.

## 2. Materials and Methods

### 2.1. Animals

Data were collected from a Holstein-Friesian herd on a commercial dairy farm near Beijing, China. The data set comprised 70,491 milking records from 484 cows over 93 consecutive days (from mid-July to mid-October). In total, 279 milking sessions were recorded (93 d × 3 milkings/d), with a mean of 254.3 ± 12.6 cows per milking session. At the start of the study, cows had a mean (±SD) parity of 2.83 ± 1.31, 122.1 ± 67.2 DIM, and a daily milk yield (DMY) of 35.01 ± 7.64 kg.

**Figure 1:**
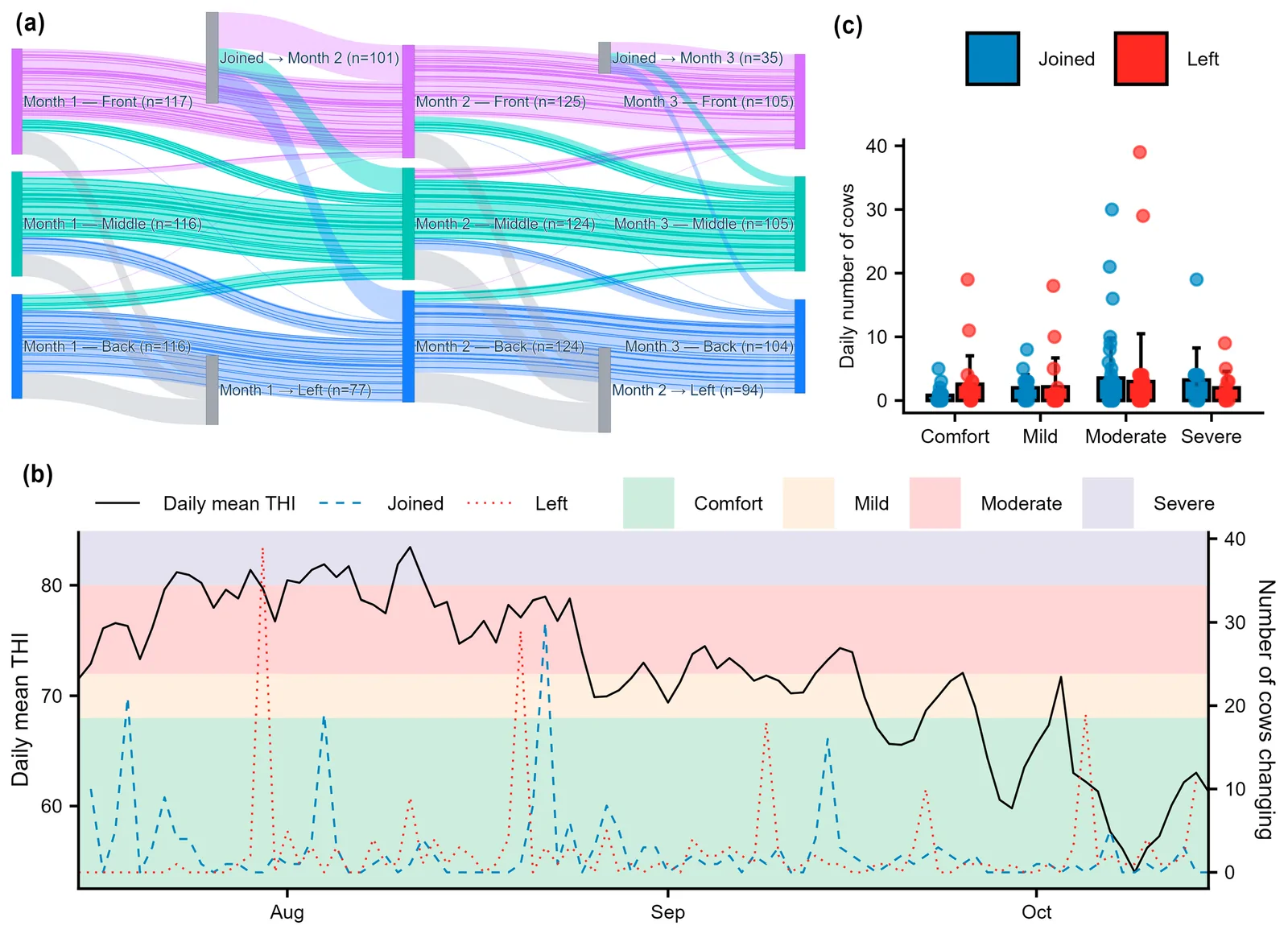
Animal and environmental background: (**a**) Monthly dynamics of the study herd over the experimental period; (**b**) thermal conditions over the entire experimental period with fluctuating herd composition; (**c**) differences in herd dynamics among thermal conditions.

Herd composition fluctuated over time due to daily management, as this barn was designated for high-producing cows and cows could be regrouped according to breeding, health status, body condition, and production status. Consequently, the number of milking records per cow varied significantly from 3 to 279. Individual cows could also shift between the front, middle, and back thirds of the milking queue (Figure 1a). Of the 484 lactating cows, 88 cows with records in at least 90% of milking sessions during the study period were selected as focal cows. This criterion was used to ensure that intraday displacement could be calculated repeatedly for the same cows across the observation period. This resulted in a total of 23,433 milking records, with a mean (±SD) parity of 2.66 ± 1.14, 140.3 ± 69.1 DIM, and a DMY of 36.83 ± 7.11 kg. Compared with cows excluded because of lower attendance (n = 396), focal cows showed no statistically detectable differences in parity or DIM, but had a higher mean DMY (36.83 ± 7.11 vs. 33.47 ± 8.74 kg/d), consistent with the management of this barn as a high-producing group.

### 2.2. Housing, Management, and Experimental Design

Cows were housed in a commercial free-stall pen (15 m × 260 m) with a concrete floor and no outdoor exercise yard, with a capacity of approximately 280 cows. A fresh total mixed ration was delivered three times daily at approximately 08:00, 14:00, and 19:00 h. Cows had ad libitum access to feed and drinking water.

Cows were milked three times daily in a 2 × 48 parallel milking parlor (ALPRO^®^ system, DeLaval, Tumba, Sweden) at approximately 09:00, 16:10, and 22:40 h, with each milking session lasting about 40 min. Cows moved voluntarily from the barn to the parlor. Before milking, the whole group entered a holding area adjacent to the parlor, where cows could move freely. The milking order for each cow was determined from automatically recorded milking times. For each milking session, the raw physical rank was numbered from 1 for the first cow to N for the last cow milked. For analyses of milking-order position, this rank was standardized by dividing it by the total number of cows milked in that session, such that values increased from earlier to later positions and the last cow had a value of 1. While being milked, cows were measured for panting score (PS) on a scale of 0–4.5, strictly following [16]. Each cow was observed for 10 s from a distance of approximately 10 m. The records from two trained observers (intraclass correlation coefficient: 0.95) were averaged. The two observers were blinded to each other’s records. PS measurement was only performed during daytime milking, that is, during the first and second daily milking sessions. Milk yields were obtained directly from the milking system. In addition, monthly Dairy Herd Improvement records were obtained for all cows. This included milk fat and protein concentrations (%), as well as SCC (×10^3^ cells/mL).

Ambient temperature (Ta) and relative humidity (RH) were recorded every 10 min using six temperature–humidity data loggers (RC-4, Elitech, Xuzhou, China; Ta accuracy ±0.1 °C, RH accuracy ±3%) evenly distributed along the pen. At each 10-min recording time, Ta and RH values were averaged across the six in-barn loggers to characterize the herd-level thermal environment within the pen. THI was calculated from these barn-average Ta and RH values using the NRC [17] formula, as expressed in Equation (1). Daily mean and maximum Ta and THI were then calculated from the resulting 10-min barn-average values. The holding area was not separately instrumented because the environmental exposure in this study was defined as the overall daily in-barn thermal environment experienced by the herd, rather than the short-term microenvironmental conditions before or during individual milking sessions.

(1) $$THI=\left(1.8\times Ta+32\right)-\left[\left(0.55-0.0055\times \mathrm{RH}\right)\times \left(1.8\times Ta-26\right)\right]$$

As shown in Figure 1b, test days were classified into comfort, mild, moderate, and severe heat stress according to daily mean THI values of <68, 68 to <72, 72 to <80, and ≥80, respectively. These thresholds were adapted from Collier et al. [18] and were used to characterize the overall daily in-barn heat-load category for the herd. The daily numbers of cows joining or leaving were compared across heat-stress conditions using non-parametric Kruskal–Wallis tests with the kruskal.test function, followed, where appropriate, by pairwise Wilcoxon rank-sum tests with the pairwise.wilcox.test function (Benjamini–Hochberg correction for multiple comparisons).

### 2.3. Statistical Analysis of Intraday Milking-Order Displacement

First, the actual full-herd order was used to quantify intraday physical displacement, which reflects the overall drift of the queue along the parlor. For each day d, let $N_{d}$ be the total number of cows milked in the parlor, and let $p_{i,d}^{S1}$, $p_{i,d}^{S2}$, and $p_{i,d}^{S3}$ denote the physical positions (1 = first in queue, $N_{d}$ = last) of a focal cow i at the first (S1), second (S2), and third (S3) milking sessions, respectively. The cow-level physical displacement on day d was then calculated by Equation (2):

(2) $$\Delta_{i,d}^{phys}\;=\;\frac{|p_{i,d}^{S1}-p_{i,d}^{S2}|+|p_{i,d}^{S2}-p_{i,d}^{S3}|}{2N_{d}}$$

so that values close to 0 indicate that a cow retained a similar physical place in the queue, whereas larger values indicate greater within-day drift along the parlor. For each day, group-level intraday physical displacement was defined as the mean of $\Delta_{i,d}^{phys}$ across all focal cows present at both milkings.

Second, intraday relative displacement was used to characterize the within-group topology of queue positions, focusing on changes in each cow’s position relative to her stable herdmates, independent of cows entering or leaving the milking herd. Specifically, for each day d, only cows in the focal group that were milked in both sessions were considered. Let $r_{i,d}^{S1}$, $r_{i,d}^{S2}$, and $r_{i,d}^{S3}$ denote the ranks of cow i among the focal cows at the first (S1), second (S2), and third (S3) milkings, respectively (1 = earliest, $n_{d}$ = latest within the focal group, where $n_{d}$ is the number of focal cows that day). The cow-level intraday relative displacement was defined by Equation (3):

(3) $$\Delta_{i,d}^{rel}\;=\;\frac{|r_{i,d}^{S1}-r_{i,d}^{S2}|+|r_{i,d}^{S2}-r_{i,d}^{S3}|}{2n_{d}}$$

which measures the extent to which a cow’s relative position among her stable herdmates changed across the three milkings. Group-level intraday relative displacement was then calculated as the mean of $\Delta_{i,d}^{rel}$ across all focal cows. The higher the value, the greater the internal reordering.

Together, intraday physical displacement captures the overall drift of the focal group along the physical queue, whereas intraday relative displacement captures re-ranking within the focal group after removing the influence of cows entering or leaving the milking herd.

Group-level intraday displacement indices were then compared across heat-stress conditions using Kruskal–Wallis tests with the kruskal.test function, followed, where appropriate, by pairwise Wilcoxon rank-sum tests with the pairwise.wilcox.test function (Benjamini–Hochberg correction for multiple comparisons). In addition, to quantify the association between milking-order displacement and thermal conditions, correlation matrices among group-level intraday displacement indices, daily THI variables, and daily Ta variables were calculated using both Pearson and Spearman’s rank methods with the cor function.

### 2.4. Statistical Analysis of Cow-Level Heterogeneity in Thermal Sensitivity of Milking Order

To characterize between-cow heterogeneity in thermal sensitivity of milking order, linear mixed-effects models were fitted to the subset of focal cows with Equation (4):

(4) $${\mathrm{MO}}_{ijk}=b_{0i}+b_{1i}{\mathrm{ENV}}_{ijk}+d_{k}+\gamma_{j}+\varepsilon_{ijk}$$

where ${\mathrm{MO}}_{ijk}$ is the milking order of cow i at session j on day k, ${\mathrm{ENV}}_{ijk}$ is the corresponding daily mean Ta, $b_{0i}$ and $b_{1i}$ are cow-specific random intercept and slope, $d_{k}$ is a random day effect capturing between-day shifts in overall queue position, $\gamma_{j}$ is a fixed effect of milking session (factor with three levels), and $\varepsilon_{ijk}$ is the residual error. Ta was entered on its original scale and was neither centered nor standardized. Therefore, the intercept corresponds to the modelled milking-order value at Ta = 0 °C and was treated as a mathematical coefficient rather than as a biologically interpretable queue position. Random intercepts and random Ta slopes were allowed to covary through an unstructured random-effects covariance matrix. Models were fitted using the lmer function of the lme4 package with restricted maximum likelihood. The model included 23,433 milking-session records from 88 focal cows, corresponding to a mean of 266.3 observations per cow.

Cow-specific random intercepts and Ta slopes ($b_{0i}$, $b_{1i}$) were extracted and standardized before clustering. K-means clustering with k = 2 was used to summarize cows with contrasting milking-order changes with increasing Ta. The two-cluster solution was motivated by the expectation that cows might show opposite directional patterns, moving relatively earlier or later in the milking queue as Ta increased. As an internal validity check, mean silhouette width was compared across solutions with k values from 2 to 6. The k = 2 solution showed the highest mean silhouette width and was therefore retained for interpretation (Table A1). Clustering was performed in the two-dimensional coefficient space using the kmeans function of the stats package.

**Figure A1:**
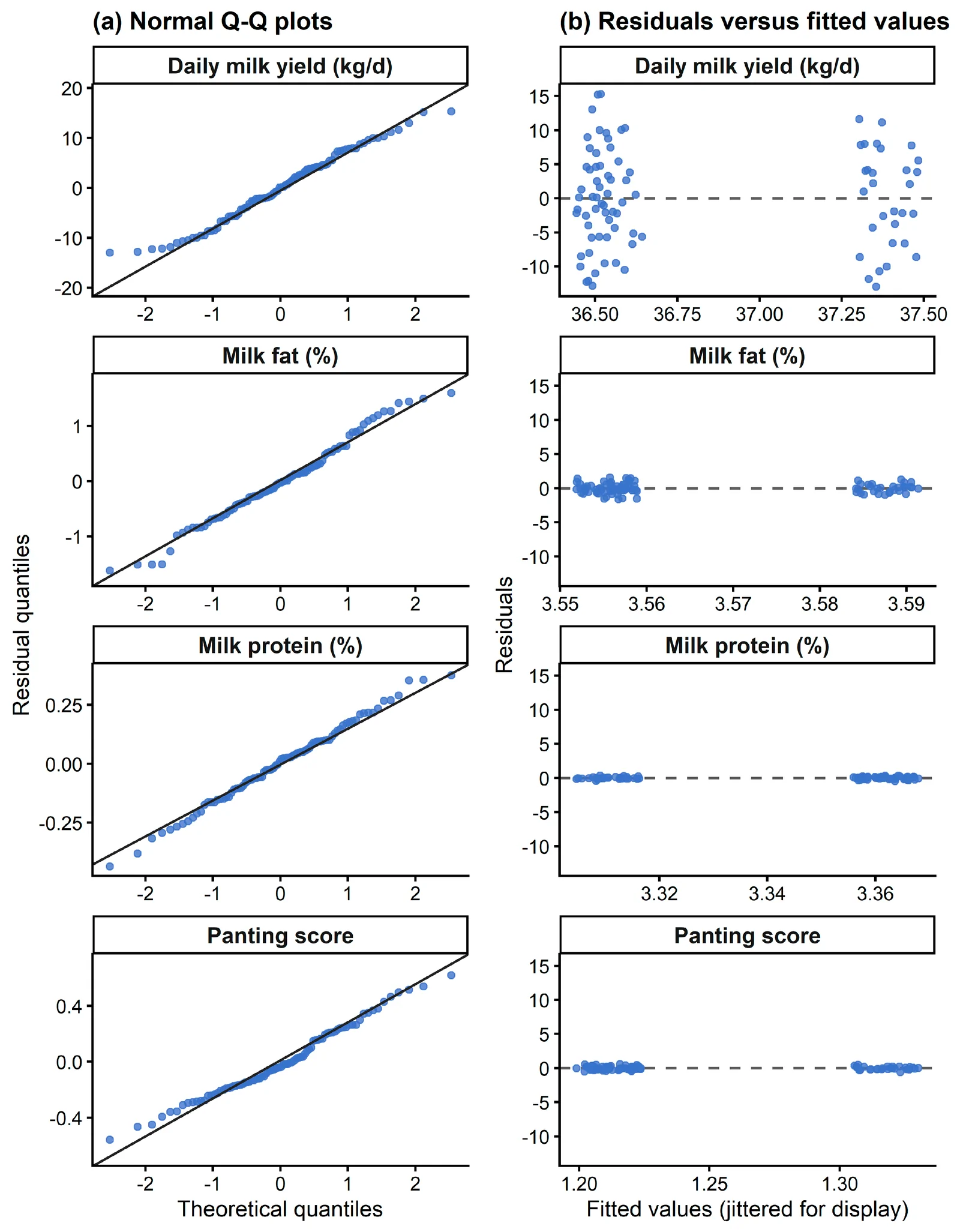
Residual diagnostics for ANOVA models used in cluster comparisons.

To evaluate how these clusters differed in cow-level characteristics, DMY observations outside the 1.5 × interquartile range (IQR) were excluded from the focal-cow dataset before cow-level traits were summarized. The lower and upper limits were 11.0 and 59.8 kg/d, respectively. This excluded 39 observations from 4 of the 88 focal cows. Residual normality and homoscedasticity were initially screened using Shapiro–Wilk and Levene’s tests, respectively. Characteristics that did not show evidence of departures from these assumptions were analyzed using one-way ANOVA, followed by Tukey’s HSD post hoc test where appropriate. Characteristics for which either assumption was not met were analyzed using Kruskal–Wallis tests, followed by Dunn’s tests with Benjamini–Hochberg adjustment where appropriate. Based on this screening, PS, DMY, milk protein, and milk fat were analyzed using one-way ANOVA, whereas parity, DIM, and SCC were analyzed using Kruskal–Wallis tests. For the four characteristics analyzed using ANOVA, Shapiro–Wilk tests did not indicate departures from residual normality (all *p* ≥ 0.186), and Levene’s tests did not indicate heteroscedasticity (all *p* ≥ 0.280). Normal Q-Q plots and residual-versus-fitted plots provided consistent graphical support for these assessments, with no pronounced systematic departures from the reference line and no obvious difference in residual spread between the two fitted group means (Figure A1). As a sensitivity analysis, cow-level traits were recalculated after retaining all original records and compared between the existing clusters using the same statistical test applied to the respective trait in the primary analysis. The overall pattern of cluster differences was not materially affected by retention of the DMY observations excluded by the IQR rule (Table A2). Results were summarized as means ± SEM.

### 2.5. Statistical Analysis of Milking-Order Stabilization After Herd Entry in Newly Introduced Cows

For cows newly introduced into the herd during the experimental period, the number of milking events required to reach a stable position in the queue was quantified, and its association with heat-stress exposure at herd entry was evaluated. A biologically meaningful stability threshold was defined by first describing the distribution of milking-order variability among focal cows under thermoneutral conditions. Specifically, for focal cows on thermally comfortable days (i.e., daily mean THI < 68), cow-level physical displacement of milking order across events was calculated, and the median of this distribution was taken as the stability threshold. For each new cow, the events-to-stabilization time was then defined as the earliest event index at which the rolling 3-event physical displacement fell at or below this threshold. Cows that did not meet this criterion by the end of the study were treated as right-censored at their last observed event.

For each newly introduced cow, her initial heat-stress exposure at herd entry was quantified using the daily mean THI on the day of first appearance, and classified into the same four levels described above. Time-to-stabilization was analyzed using Kaplan–Meier survival curves stratified by entry heat-stress exposure, estimated with the survfit function from the survival package. The differences between curves were tested by log-rank tests using the survdiff function. In addition, Cox proportional hazards models were fitted with the coxph function to estimate hazard ratios (HR) for stabilization across four-level heat-stress exposure, with and without adjustment for cow-level covariates such as parity and DIM. Time was measured as the number of milking events to stabilization. Cows reaching the stabilization criterion at the same event time were treated as tied event times. Cox proportional hazards models were used because they accommodate right censoring while estimating the association between covariates and the event-specific hazard of reaching stabilization. Tied event times were handled using Efron’s approximation. Baseline covariates were taken from the first record of each new cow. DIM was classified as 1–100 (reference), 101–200, and ≥201. Parity was classified as 1, 2, and ≥3 lactations. DMY was classified into low, medium, and high classes based on tertiles at 27.40 and 37.07 kg. Proportional hazards assumptions were checked using Schoenfeld residuals computed with the cox.zph function.

## 3. Results and Discussion

To the best of our knowledge, this is the first study to investigate the association between heat stress and milking-order displacement. Across the 93 test days, daily mean THI ranged from 54.0 to 83.5, with an overall mean ± SD of 72.3 ± 7.1 (Figure 1b). Using the predefined thresholds, 22 d (23.7%) were classified as comfort, 19 d (20.4%) as mild heat stress, 39 d (41.9%) as moderate heat stress, and 13 d (14.0%) as severe heat stress. Notably, the number of cows joining or leaving the herd did not differ significantly across test days classified by thermal conditions (Figure 1c). Because regrouping-related stress may affect social behavior [19], herd movements were considered as a potential source of confounding. Their similar distribution across thermal categories reduces concern that daily herd movements were strongly imbalanced between conditions, although residual confounding cannot be excluded.

### 3.1. Intraday Milking-Order Displacement and Associations with Heat Stress

**Figure 2:**
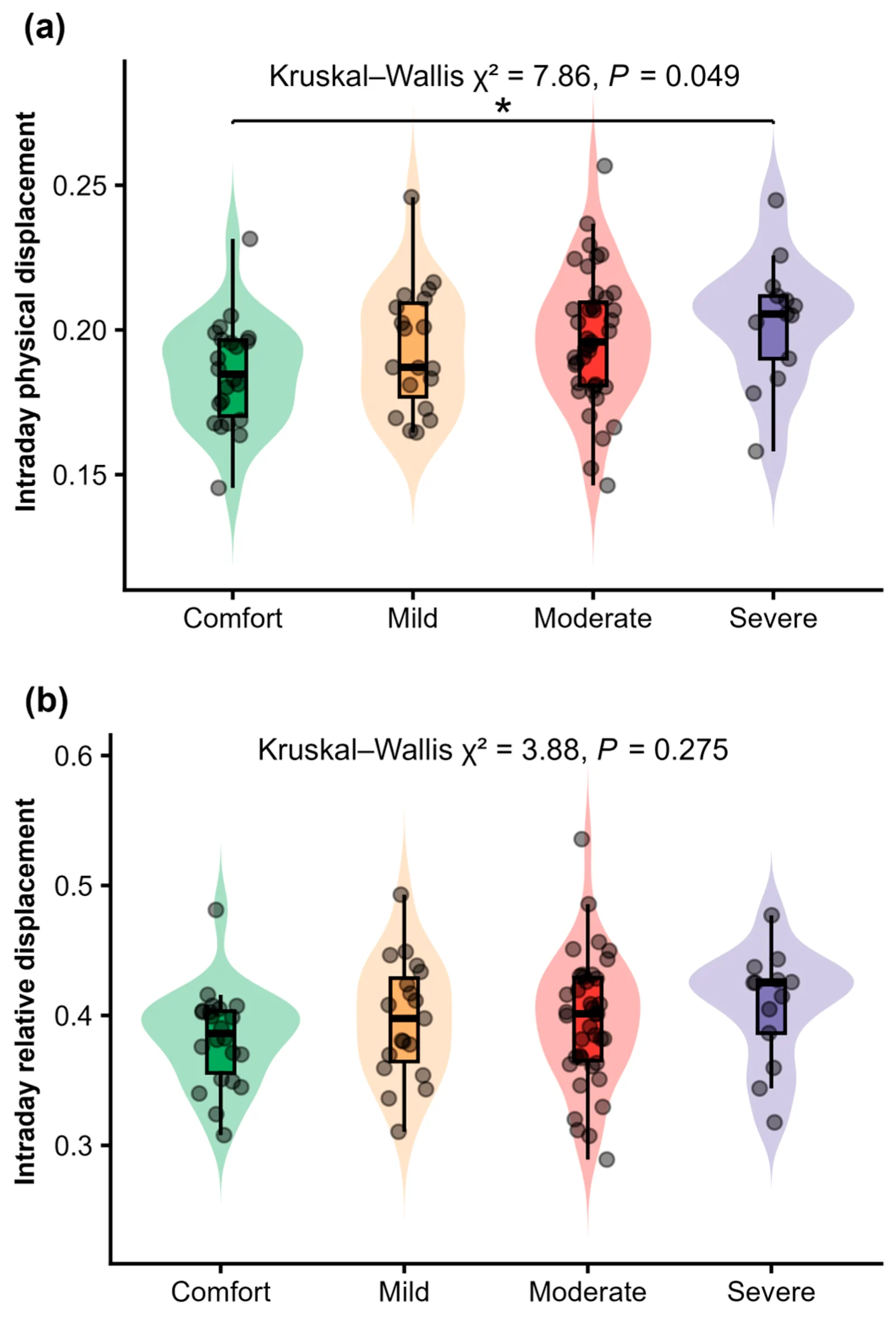
Group-level intraday (**a**) physical and (**b**) relative displacement indices across four heat-stress conditions (n = 93 test days). The lower edge, midline, and upper edge of each box represent the 25th, 50th, and 75th percentiles, respectively. * indicates *p* < 0.05.

The intraday physical displacement index showed modest differences across 93 test days classified into four-level heat-stress exposure (*p* = 0.049; Figure 2a). Median (IQR) values increased from 0.185 (0.170–0.196) on comfort days to 0.187 (0.177–0.209) on mild days, 0.196 (0.181–0.210) on moderate days, and 0.206 (0.190–0.212) on severe days. Post hoc pairwise Wilcoxon tests identified greater intraday physical displacement on severe days than on comfort days (*p* = 0.049; Figure 2a). For the intraday relative displacement index, medians (IQR) show an increasing trend of 0.386 (0.356–0.403), 0.398 (0.365–0.429), 0.401 (0.365–0.429), and 0.425 (0.386–0.427) on comfort, mild, moderate, and severe days, respectively (Figure 2b). However, the overall Kruskal–Wallis test across the four categories was not statistically significant (*p* = 0.275), and no pairwise post hoc comparisons were performed.

**Figure 3:**
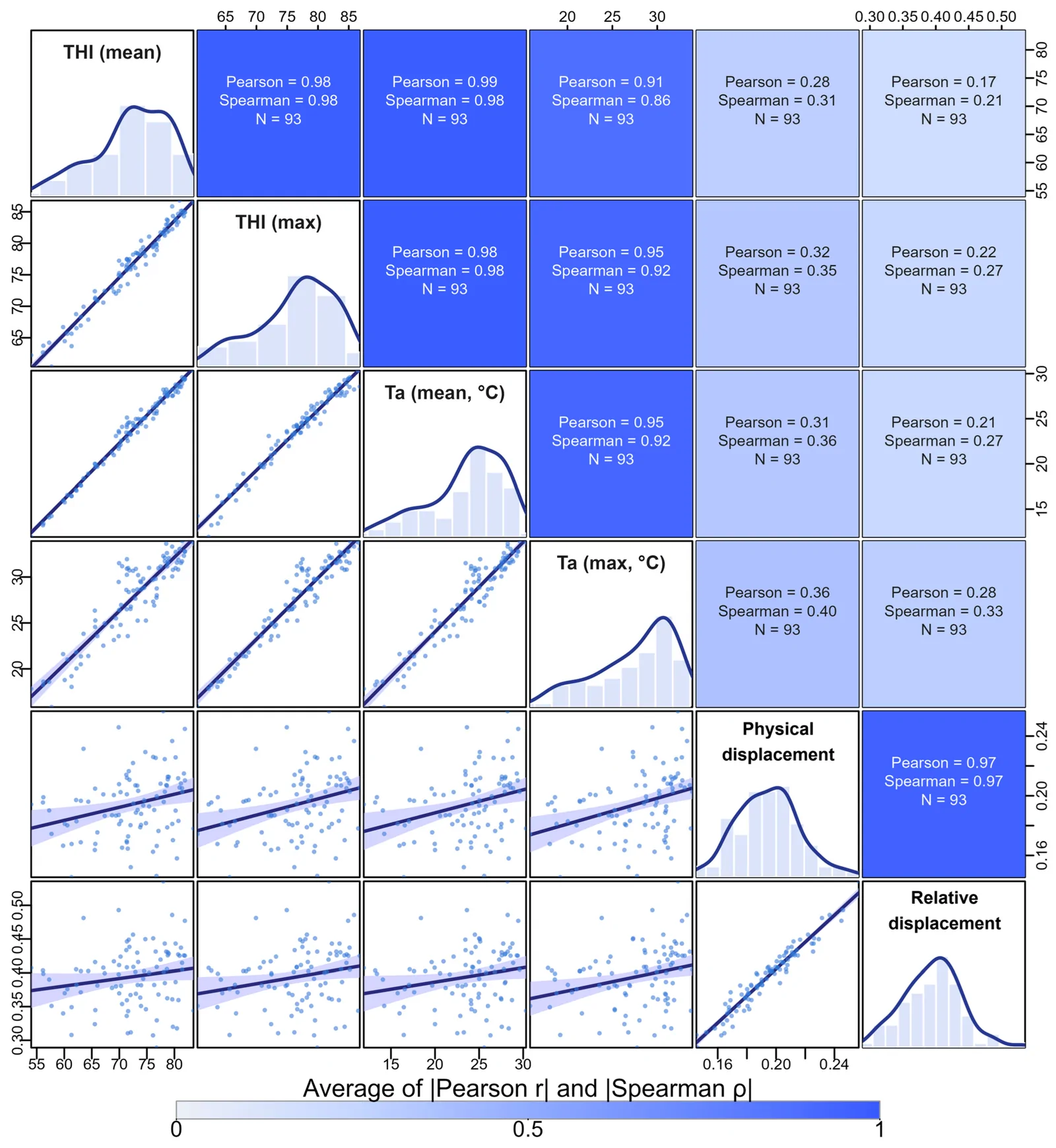
Correlations between group-level intraday displacement indices, daily mean and maximum THI, and daily mean and maximum Ta (n = 93 test days).

In the daily correlation analysis (Figure 3), both daily mean THI and daily mean Ta were positively associated with intraday displacement indices, with consistently stronger correlations for physical displacement than for relative displacement. In addition, daily mean Ta always had stronger correlations with intraday displacement indices compared with daily mean THI in our dataset. This result is consistent with previous dairy studies conducted in similar temperate continental climates, where Ta also had better associations with physiological, behavioral, and production traits than THI [6,20]. Therefore, the daily mean Ta was further used for exploring cow-level sensitivity to heat stress in the next section.

Taken together, these results indicate a modest increase in within-day milking-order displacement as daily heat load rose. The fact that this effect was more pronounced for absolute physical positions than for relative positions within the focal group is informative, because both indices were computed on exactly the same core cows but differ in how they treat the rest of the herd. The physical index preserves each core cow’s observed rank in the full queue, whereas the relative index reorders only the core cows and thus removes the influence of non-core animals inserting before, between, or after them. Therefore, these results indicate increased reordering of non-core cows around a comparatively stable hierarchy of core cows, rather than a wholesale reshuffling of the hierarchy within the focal group itself. A plausible interpretation is that cows that are not yet firmly anchored in the herd’s dominance structure may be more susceptible in their milking-order response to heat stress, repeatedly changing how they “slot in” around core animals under hotter conditions [21].

These findings align with broader evidence that heat stress reshapes dairy cow behavior, including altered time budgets, increased standing and shade-seeking, and changes in social interactions and competitive behaviors around key resources [22,23,24]. Previous work on milking-order consistency in housed herds has largely characterized order as moderately stable over weeks to months, with most cows varying within a limited band of their preferred position across days [15]. Our day-level analyses extend this literature by showing that even within a single day, the degree of reordering between successive milkings is not fixed but covaries with heat stress. At the same time, the modest effect sizes and only one significant pairwise contrast (between severe and comfort days) emphasize that milking-order stability is relatively robust to mild and moderate heat loads and only begins to show detectable perturbation under the most stressful thermal conditions.

### 3.2. Cow-Level Heterogeneity in Thermal Sensitivity of Milking Order

**Figure 4:**
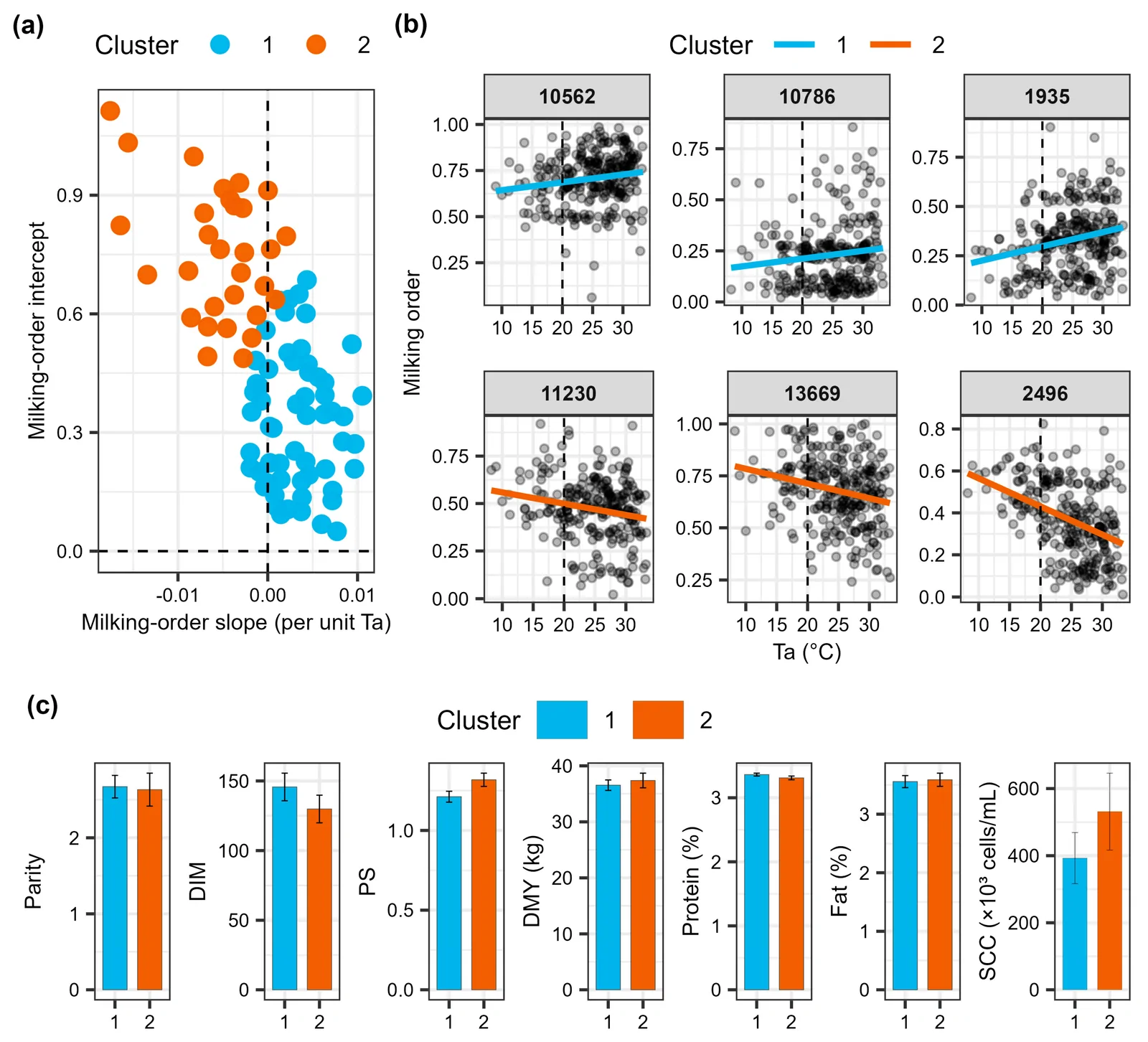
Cow-level heterogeneity in thermal sensitivity of milking order (n = 88 core cows). (**a**) Clusters of individual milking order versus ambient temperature (Ta) slopes and intercepts among core cows. Because Ta was not centered, intercepts correspond to the modelled value at 0 °C and are shown only as part of the clustering display. (**b**) Representative cow-level trajectories for each cluster across the observed temperature range. (**c**) Cluster differences in parity, DIM, PS, DMY (kg), milk protein and fat (%), and SCC (×10^3^ cells/mL), with error bars indicating SEM.

Using the exploratory two-cluster solution based on cow-specific coefficients linking Ta to milking order, 88 focal cows were summarized into two descriptive clusters (Figure 4a–c). Cluster 1 (n = 58) comprised cows that tended to occupy earlier positions in the queue and showed slightly increasing milking-order positions as Ta rose, whereas Cluster 2 (n = 30) comprised cows that tended to occupy later positions and showed decreasing milking-order positions with increasing Ta (Figure 4a). Representative trajectories from three cows per cluster illustrate that these patterns were expressed consistently across the observed temperature range of approximately 8 to 33 °C (Figure 4b).

Cluster membership was only weakly associated with recorded cow-level traits (Figure 4c). Parity did not differ between clusters (Cluster 1: 2.7 ± 0.15; Cluster 2: 2.6 ± 0.22; *p* = 0.79). DIM showed similar distributions (Cluster 1: 145.7 ± 9.92; Cluster 2: 129.9 ± 9.92; *p* = 0.36). Although parity and lactation stage have been associated with absolute parlor-entry order in other herds [11], they were not associated with cluster membership in the present analysis. Milk yield and composition were comparable across clusters (DMY: 36.5 ± 0.94 vs. 37.4 ± 1.31 kg, *p* = 0.602; protein: 3.4 ± 0.02 vs. 3.3 ± 0.03%, *p* = 0.181; fat: 3.6 ± 0.10 vs. 3.6 ± 0.11%, *p* = 0.847). SCC was numerically higher in Cluster 2 (392.7 ± 76.12 vs. 531.4 ± 114.66 ×10^3^ cells/mL) but not statistically different (*p* = 0.146). There was no statistically significant difference in PS, but it was close to significant, with an average of 1.2 ± 0.03 in Cluster 1 and 1.3 ± 0.04 in Cluster 2 (*p* = 0.063). PS is widely used as a practical and biologically meaningful indicator of heat load in cattle [25,26]. Previous studies under comparable thermal conditions show that cattle with higher PS also maintain elevated respiration rate and body temperature compared with more heat-tolerant cows [27,28]. Thus, the higher PS observed in Cluster 2 may suggest greater physiological heat strain. However, this trend alone does not establish Cluster 2 as a heat-susceptible group and should be interpreted cautiously.

**Figure 5:**
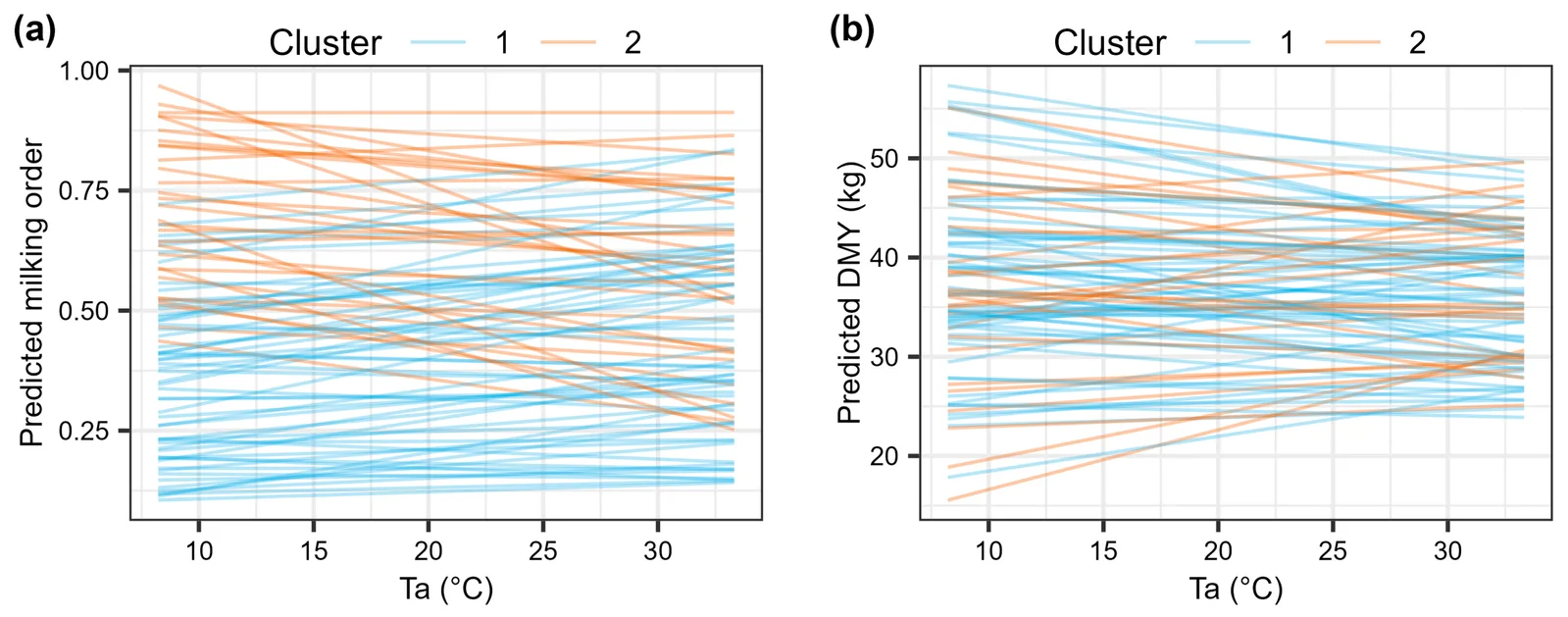
Cow-level (**a**) milking order trajectories and (**b**) DMY trajectories as a function of Ta, stratified by milking order-based clusters. Lines represent cow-specific fitted trajectories from linear mixed-effects models.

The visual convergence of milk yields under hot conditions shown in Figure 5 is consistent with previous works that high-producing cows often experience steeper yield losses as heat stress increases, thereby narrowing production differences among cows [29,30]. However, cows grouped into clusters based on their milking-order trajectories in response to heat stress did not show a corresponding separation in their DMY to Ta slopes, and milking-order slopes explained only a small fraction of the variance in milk-yield slopes (R^2^ = 0.058). This suggests that the change in milking order with increasing Ta is not simply a by-product of how milk yield responds to heat stress, but contains far more information than just the loss in milk yield itself.

One possible behavioral interpretation is that the overall in-barn heat-load background may be associated with subsequent queuing behavior. Because daily mean Ta and THI were derived from sensors distributed along the pen, these variables characterize the thermal conditions experienced by the herd before cows entered the holding area and parlor. Previous studies have shown that heat stress can alter activity and social behavior and may intensify competition for key resources, such as water and shade [5,31,32]. Increased competition may also affect dominance-related access to shared resources in dairy cows [33]. Therefore, under hotter in-barn conditions, changes in activity, social interactions, or motivation to move through the milking process may contribute to changes in queue position. However, holding-area and parlor microenvironments, individual cooling use, and competitive interactions at the parlor entrance were not measured in the present study. These mechanisms should therefore be regarded as hypotheses rather than direct explanations of the observed associations.

### 3.3. Milking-Order Stabilization After Herd Entry in Newly Introduced Cows

**Figure 6:**
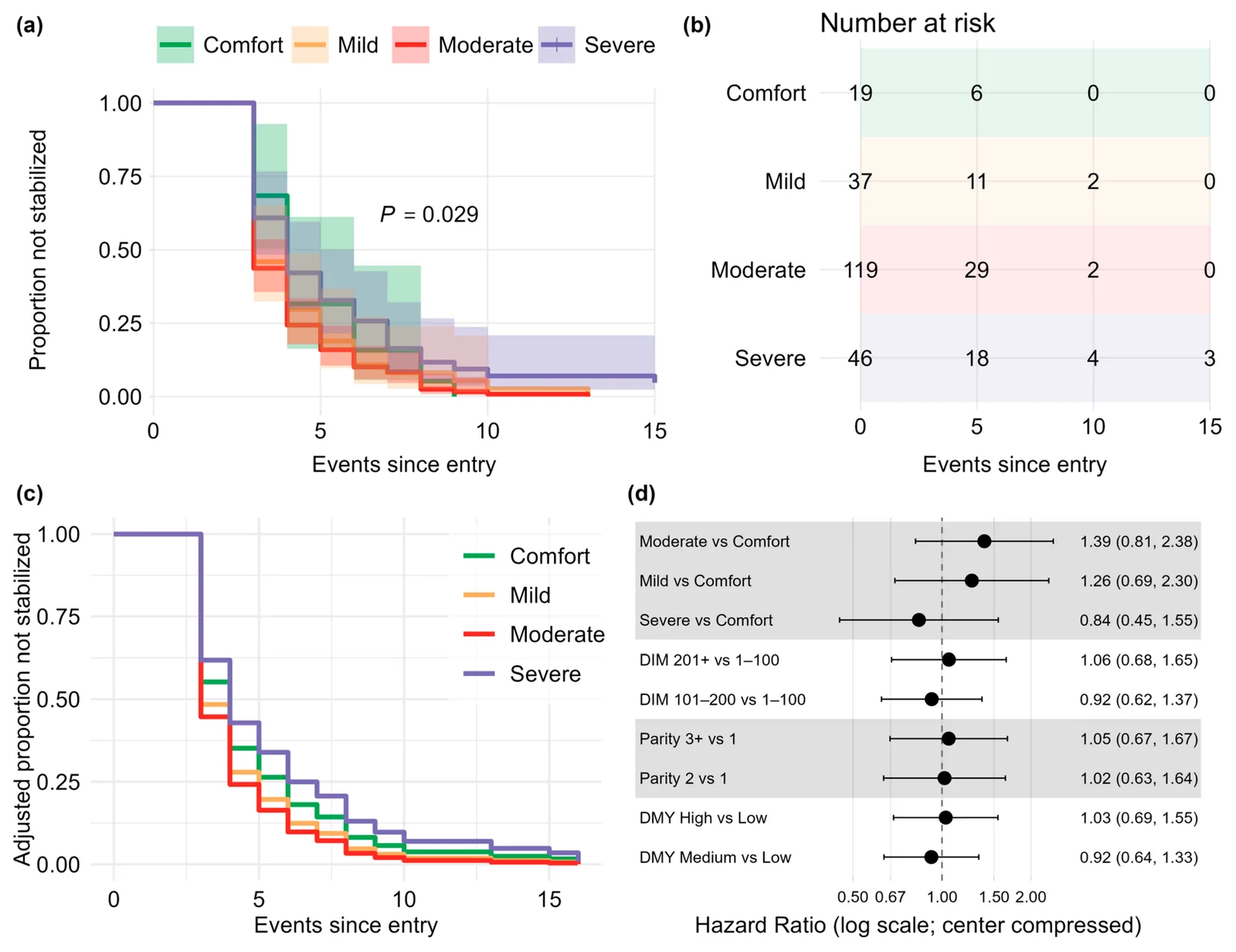
Time to milking order stabilization after herd entry by initial four-level heat-stress exposure (n = 221 newly introduced cows). (**a**) Kaplan–Meier curves for time to stabilization. (**b**) Number of cows at risk at each event time. (**c**) Cox model-adjusted survival curves for time to stabilization. (**d**) Forest plot of adjusted HR (95% CI) for DIM, parity, and DMY from the same multivariable Cox model. Points indicate HR estimates and horizontal lines indicate 95% CIs. The dashed vertical line indicates HR = 1.

The stabilization threshold was set at 0.159, corresponding to the median 3-event physical displacement calculated from the focal group under thermally comfortable conditions. Among 221 newly introduced cows with usable data, 219 reached a predefined milking-order stabilization event during follow-up, and the remaining two were censored. Kaplan–Meier curves showed small but significant differences in the number of milking events required to reach stabilization among the initial four-level thermal exposure at herd entry (*p* = 0.029; Figure 6a,b). Median events to stabilization were 4 (95% CI 4–6) for comfort (n = 19, 19 events), 3 (95% CI 3–4) for mild (n = 37, 37 events), 3 (95% CI 3–4) for moderate (n = 119, 119 events), and 4 (95% CI 3–6) for severe (n = 46, 44 events, 2 censored) groups.

**Figure 7:**
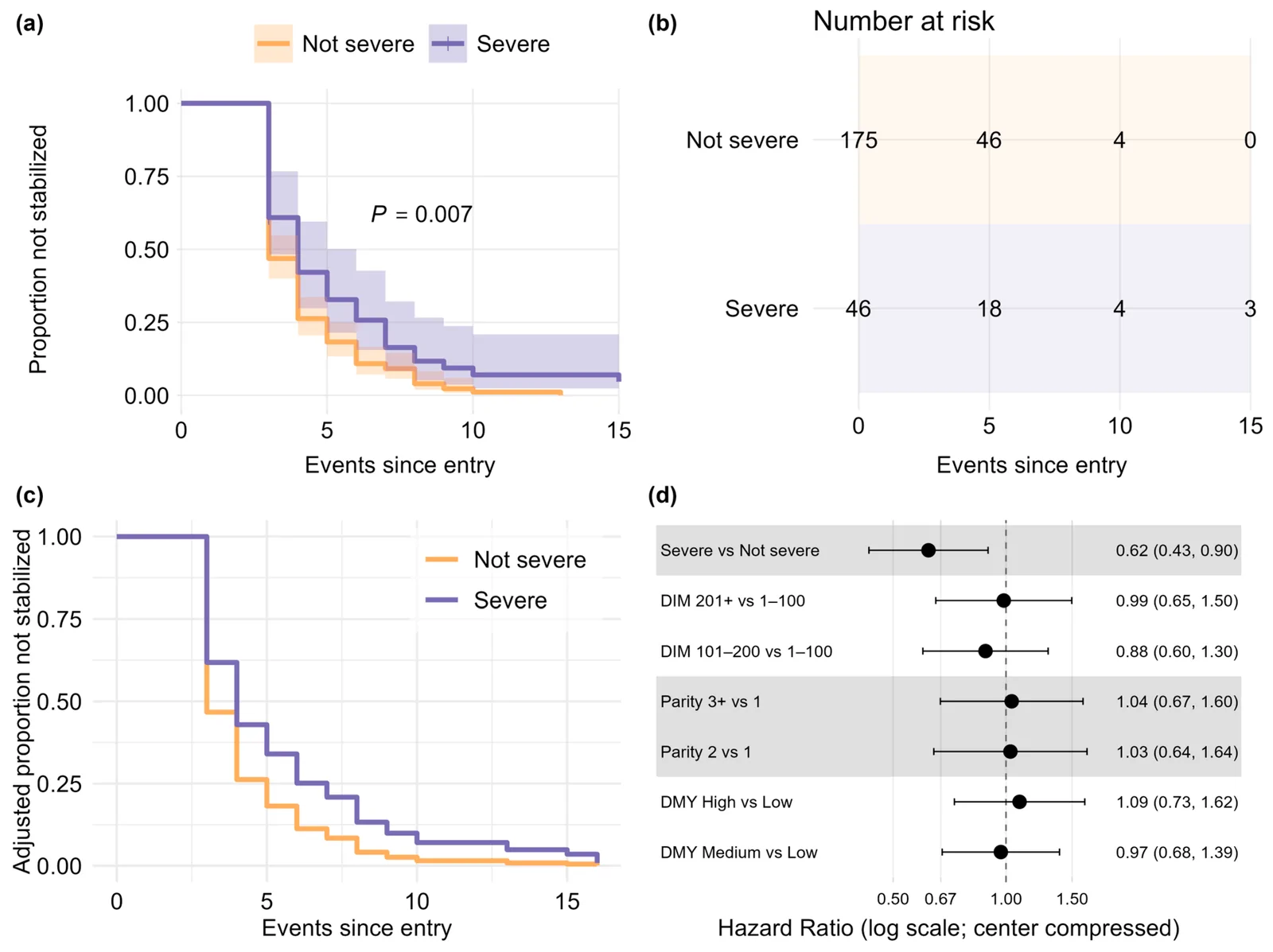
Time to milking order stabilization after herd entry by dichotomized initial heat-stress exposure (n = 221 newly introduced cows). (**a**) Kaplan–Meier curves for time to stabilization. (**b**) Number of cows at risk at each event time. (**c**) Cox model-adjusted survival curves for time to stabilization. (**d**) Forest plot of adjusted HR (95% CI) for DIM, parity, and DMY from the same multivariable Cox model. Points indicate HR estimates and horizontal lines indicate 95% CIs. The dashed vertical line indicates HR = 1.

The severe versus non-severe comparison was conducted as an exploratory secondary analysis to aid interpretation of the four-level results. In this case, the separation between survival curves became more pronounced (*p* = 0.007; Figure 7a,b). Cows entering on non-severe days (n = 175, 175 events) had a median of three milking events to stabilization (95% CI 3.0–4.0), whereas those entering under severe conditions (n = 46, 44 events, 2 censored) had a median of four events (95% CI 3–6).

We further examined whether the above-detected differences persisted after accounting for cow-level covariates by using adjusted Cox proportional hazards models (all *p* ≥ 0.332). The global proportional hazards test did not provide evidence of non-proportional hazards for any covariates, thus allowing for Cox modeling. However, in the four-level model, there was no clear association between heat-stress exposures at herd entry and time to stabilization after adjustment for covariates (all *p* ≥ 0.228; Figure 6c,d).

In the binary model, initial severe exposure was associated with a lower hazard of stabilization compared with non-severe exposure after adjustment (HR 0.62, 95% CI 0.43–0.90, *p* = 0.011; Figure 7c,d). This indicates a 38% lower instantaneous hazard of reaching the predefined stabilization criterion among cows entering under severe conditions. Covariate effects remained small and not significant (all *p* ≥ 0.521). The proportional hazards assumption also held for this model (all *p* ≥ 0.292). Since the adjusted four-level model did not show clear associations across all thermal categories, these findings should be interpreted as an exploratory association rather than evidence of a general management threshold.

Regrouping has been repeatedly shown to induce social stress, increase behavioral variability, and transiently affect activity, rumination, and milk yield in housed dairy cows [19,34,35]. Heat stress itself also intensifies competition for heat-abatement resources such as water and shade, and alters how cows of different social status access these resources [31]. Our results indicate that the daily in-barn thermal context at herd entry was associated with subsequent milking-order stabilization in the exploratory binary comparison. Because the study did not measure holding-area conditions, cooling use, or social interactions at the parlor entrance, the behavioral pathways underlying this association remain uncertain. Future studies should combine barn-level thermal monitoring with measurements of holding-area microenvironment, cooling use, and social interactions to determine whether these factors contribute to stabilization after herd entry.

### 3.4. Strengths, Limitations, and Future Perspectives

A key strength of this study is the multi-layered description of milking-order dynamics under heat stress. By combining herd-level intraday displacement, cow-level thermal sensitivity clusters, and entry-level time to stabilization, we link immediate queue variability, persistent individual differences, and the integration trajectory of newly introduced cows within a single behavioral framework. This approach aligns with recent developments in precision livestock farming that emphasize using routinely collected data streams (e.g., parlor logs) as digital phenotypes for behavior and welfare.

Several limitations should be considered. The study was conducted in a single high-yielding housed herd in a temperate continental climate, which constrains generalizability across breeds, housing systems, and climatic zones. Recent work has shown that the magnitude and timing of heat-stress impacts on milk production and behavior in temperate regions depend strongly on local microclimates [20,36,37]. In addition, the 93 test days represented repeated observations within a single study period. Temporal autocorrelation and seasonal patterns may therefore have contributed to the observed day-level associations and were not explicitly modelled in the non-parametric day-level comparisons. The focal-cow analysis was also restricted to cows with records in at least 90% of milking sessions. Although parity and DIM did not differ detectably between included and excluded cows, the included cows had a higher mean DMY. Thus, the focal-cow findings may be less representative of cows with intermittent milking records or lower milk yield. Moreover, heat stress was characterized using the daily in-barn thermal environment, based on Ta and RH measurements averaged across six loggers distributed along the pen. This approach was intended to represent the herd’s overall daily heat-load background, but it did not quantify individual thermal exposure or short-term microenvironmental conditions experienced in the holding area and parlor. Accordingly, the THI categories used in this study should be regarded as context-specific operational groupings rather than universal biological thresholds. Comparative evaluations in lactating cows indicate that the relative performance of thermal indices may differ across environmental settings and physiological outcomes [38]. Finally, dominance rank, social networks, and detailed agonistic or affiliative interactions around the parlor entrance were not recorded, even though recent work indicates that dedicated distance-based sensing can capture social interactions that are not fully reflected in queue-based measures [39].

These limitations suggest clear priorities for future research. Multi-herd, multi-season studies across diverse climatic regions and parlor designs are needed to test whether the pattern observed here replicates under different management conditions. Integration of milking-order trajectories with richer precision livestock farming techniques, including wearables- and vision-based tracking of social interactions, would allow more explicit modelling of how competition, affiliative relationships, and heat stress jointly shape queue behavior. Therefore, in the future multi-group cattle research, it is necessary to test whether the management intervention measures are effective (including cattle admission time and cooling measures before admission), and to carry out such research before formulating specific management recommendations.

## 4. Conclusions

In this observational study, daily heat load was associated with milking-order dynamics and stabilization after herd entry in a large commercial dairy herd. Within-day milking-order displacement at the herd level increased modestly between thermally comfortable and severely hot days, whereas queuing patterns remained comparatively stable under mild and moderate heat stress. Cows expressed consistent individual differences in how their milking position changed with ambient temperature that were largely independent of parity, DIM, DMY, and SCC, with only a weak association with panting score. Among newly introduced cows, the adjusted four-level analysis did not show a clear association between initial thermal category and time to stabilization. In an exploratory binary comparison, cows entering under severe conditions had a lower hazard of reaching the predefined stabilization criterion than cows entering under non-severe conditions. These findings indicate that milking-order patterns derived from routine parlor logs may provide a behavioral indicator associated with daily heat load. However, the underlying behavioral mechanisms were not measured directly, and multi-herd intervention studies are needed before recommendations regarding the timing of herd entry under severe heat conditions can be made.

## Data Availability

The original contributions presented in this study are included in the article. Further inquiries can be directed to the corresponding author.

## Abbreviations

The following abbreviations are used in this manuscript:

DMY	Daily milk yield.
HR	Hazard ratio.
IQR	Interquartile range.
PS	Panting score.
RH	Relative humidity.
Ta	Ambient temperature.
THI	Temperature–humidity index.

## Appendix A

**Table A1:** Internal validity indices for k-means solutions.

Number of Clusters (k)	Mean Silhouette Width
2	0.471
3	0.370
4	0.354
5	0.378
6	0.388

**Table A2:** Sensitivity analysis of cluster differences in cow-level characteristics with and without exclusion of DMY outliers. Values are *p* values from the primary between-cluster tests.

Trait	Outliers Excluded (23,433 Records from 88 Cows)	All Records (23,472 Records from 88 Cows)
Parity	0.794	0.794
DIM	0.362	0.358
PS	0.063	0.064
DMY	0.602	0.618
Protein	0.181	0.179
Fat	0.847	0.848
SCC	0.148	0.150

## References

[1] Palandri C., Frank E.G., Kimhi A., Lavon Y., Ezra E., Fishman R. (2025). High-Frequency Data Reveal Limits of Adaptation to Heat in Animal Agriculture. Sci. Adv..

[2] Becker C.A., Collier R.J., Stone A.E. (2020). Invited review: Physiological and Behavioral Effects of Heat Stress in Dairy Cows. J. Dairy Sci..

[3] Burhans W.S., Rossiter Burhans C.A., Baumgard L.H. (2022). Invited review: Lethal Heat Stress: The Putative Pathophysiology of a Deadly Disorder in Dairy Cattle. J. Dairy Sci..

[4] Herbut P., Hoffmann G., Angrecka S., Godyń D., Vieira F.M.C., Adamczyk K., Kupczyński R. (2021). The Effects of Heat Stress on the Behaviour of Dairy Cows—A Review. Ann. Anim. Sci..

[5] McDonald P.V., von Keyserlingk M.A.G., Weary D.M. (2020). Hot Weather Increases Competition Between Dairy Cows at the Drinker. J. Dairy Sci..

[6] Shu H., Bindelle J., Guo L., Gu X. (2023). Determining the Onset of Heat Stress in a Dairy Herd Based on Automated Behaviour Recognition. Biosyst. Eng..

[7] Beggs D.S., Jongman E.C., Hemsworth P.H., Fisher A.D. (2018). Short Communication: Milking Order Consistency of Dairy Cows in Large Australian Herds. J. Dairy Sci..

[8] Grasso F., De Rosa G., Napolitano F., Di Francia A., Bordi A. (2007). Entrance Order and Side Preference of Dairy Cows in the Milking Parlour. Ital. J. Anim. Sci..

[9] Berkhout M.J., Auldist M.J.A., Douglas M.L., Thomson A.L., Giri K., Jacobs J.L., Wright M.M. (2025). Milk Yield and Pasture Nutrient Availability Associated with Milking Order in Commercial Dairy Herds. Anim. Prod. Sci..

[10] Cullen B.R., Weng H.M., Talukder S., Cheng L. (2020). Cow Milking Order and Its Influence on Milk Production in a Pasture-Based Automatic Milking System. Anim. Prod. Sci..

[11] Hansson I., Woudstra S. (2024). Associations of Parity and Lactation Stage with the Order Cows Enter the Milking Parlor. JDS Commun..

[12] Polikarpus A., Kaart T., Mootse H., De Rosa G., Arney D. (2015). Influences of Various Factors on Cows’ Entrance Order into the Milking Parlour. Appl. Anim. Behav. Sci..

[13] Rathore A.K. (1982). Order of Cow Entry at Milking and Its Relationships with Milk Yield and Consistency of the Order. Appl. Anim. Ethol..

[14] Berry D.P., McCarthy J. (2012). Genetic and Non-Genetic Factors Associated with Milking Order in Lactating Dairy Cows. Appl. Anim. Behav. Sci..

[15] Vargas-Bello-Pérez E., Bastías-Ruz J., Toro-Mujica P., Teixeira D.L., Enriquez-Hidalgo D. (2020). Interplay between Productive Traits, the Social Rank and the Cow’s Stability in the Order of Entrance to the Milking Parlour. J. Agric. Sci..

[16] Gaughan J., Mader T., Holt S., Lisle A. (2008). A New Heat Load Index for Feedlot Cattle. J. Anim. Sci..

[17] NRC (1971). A Guide to Environmental Research on Animals.

[18] Collier R.J., Laun W.H., Rungruang S., Zimbleman R.B. (2012). Quantifying Heat Stress and Its Impact on Metabolism and Performance. Proceedings of the Florida Ruminant Nutrition Symposium, Gainesville, FL, USA, 2012.

[19] Marumo J.L., Lusseau D., Speakman J.R., Mackie M., Byar A.Y., Cartwright W., Hambly C. (2024). Behavioural Variability, Physical Activity, Rumination Time, and Milk Characteristics of Dairy Cattle in Response to Regrouping. Animal.

[20] Shu H., Guo L., Bindelle J., Fang T., Xing M., Sun F., Chen X., Zhang W., Wang W. (2022). Evaluation of Environmental and Physiological Indicators in Lactating Dairy Cows Exposed to Heat Stress. Int. J. Biometeorol..

[21] Soffié M., Thinès G., De Marneffe G. (1976). Relation between Milking Order and Dominance Value in a Group of Dairy Cows. Appl. Anim. Ethol..

[22] Jurkovich V., Hejel P., Kovács L. (2024). A Review of the Effects of Stress on Dairy Cattle Behaviour. Animals.

[23] Polsky L., Von Keyserlingk M.A.G. (2017). Invited Review: Effects of Heat Stress on Dairy Cattle Welfare. J. Dairy Sci..

[24] Vizzotto E.F., Fischer V., Thaler Neto A., Abreu A.S., Stumpf M.T., Werncke D., Schmidt F.A., McManus C.M. (2015). Access to Shade Changes Behavioral and Physiological Attributes of Dairy Cows during the Hot Season in the Subtropics. Animal.

[25] Shu H., Wang W., Guo L., Bindelle J. (2021). Recent Advances on Early Detection of Heat Strain in Dairy Cows Using Animal-Based Indicators: A Review. Animals.

[26] Wijffels G., Sullivan M., Gaughan J. (2021). Methods to Quantify Heat Stress in Ruminants: Current Status and Future Prospects. Methods.

[27] Gaughan J., Mader T. (2014). Body Temperature and Respiratory Dynamics in Un-Shaded Beef Cattle. Int. J. Biometeorol..

[28] Islam M.A., Lomax S., Doughty A.K., Islam M.R., Thomson P.C., Clark C.E.F. (2023). Revealing the Diversity of Internal Body Temperature and Panting Response for Feedlot Cattle under Environmental Thermal Stress. Sci. Rep..

[29] Carabaño M.J., Logar B., Bormann J., Minet J., Vanrobays M.L., Díaz C., Tychon B., Gengler N., Hammami H. (2016). Modeling Heat Stress under Different Environmental Conditions. J. Dairy Sci..

[30] Collier R.J., Baumgard L.H., Zimbelman R.B., Xiao Y. (2019). Heat Stress: Physiology of Acclimation and Adaptation. Anim. Front..

[31] Deniz M., Sena A.R., De-Sousa K.T., Vieira F.M.C., de Souza E.R., Hötzel M.J., Dittrich J.R. (2025). Herd Dominance Influences Dairy Cows’ Use of Heat Abatement Resources in a Silvopastoral System. Animals.

[32] Tsai Y.C., Hsu J.T., Ding S.T., Rustia D.J.A., Lin T.T. (2020). Assessment of Dairy Cow Heat Stress by Monitoring Drinking Behaviour Using an Embedded Imaging System. Biosyst. Eng..

[33] Sheng K., Foris B., Krahn J., Weary D.M., von Keyserlingk M.A.G. (2024). Redefining Dominance Calculation: Increased Competition Flattens the Dominance Hierarchy in Dairy Cows. J. Dairy Sci..

[34] Valníčková B., Bartošová J., Bartoš L. (2024). Losing a Herd Mate: Negative Effects on Milk Yield and Udder Health Indicators in Loose-Housed Dairy Cattle. Animals.

[35] von Keyserlingk M.A.G., Olenick D., Weary D.M. (2008). Acute Behavioral Effects of Regrouping Dairy Cows. J. Dairy Sci..

[36] Leandro M.A., Stock J., Bennewitz J., Chagunda M.G.G. (2024). Is Heat Stress a Growing Problem for Dairy Cattle Husbandry in the Temperate Regions? A Case Study of Baden-Württemberg in Germany. J. Anim. Sci..

[37] Leliveld L.M.C., Lovarelli D., Riva E., Provolo G. (2025). Dairy cow behaviour and physical activity as indicators of heat stress. Ital. J. Anim. Sci..

[38] Yan G., Li H., Zhao W., Shi Z. (2020). Evaluation of Thermal Indices Based on Their Relationships with Some Physiological Responses of Housed Lactating Cows under Heat Stress. Int. J. Biometeorol..

[39] Marina H., Ren K., Hansson I., Fikse W.F., Nielsen P.P., Rönnegård L. (2024). New Insight into Social Relationships in Dairy Cows and How Time of Birth, Parity, and Relatedness Affect Spatial Interactions Later in Life. J. Dairy Sci..

